# Changes in the Outcome of Pediatric Patients with Acute Lymphoblastic Leukemia—Single Center, Real-Life Experience

**DOI:** 10.3390/medicina61071129

**Published:** 2025-06-23

**Authors:** Letitia E. Radu, Andra D. Marcu, Ana M. Bica, Ana M. Marcu, Andreea N. Serbanica, Cristina G. Jercan, Cerasela Jardan, Delia C. Popa, Cristina Constantin, Andrei M. Vasilescu, Oana O. Niculita, Roxana Sfetea, Anca Colita

**Affiliations:** 1 Faculty of General Medicine, “Carol Davila” University of Medicine and Pharmacy, 020021 Bucharest, Romania; 2 Fundeni Clinical Institute, 258 Fundeni Road, 022328 Bucharest, Romania; 3 Politehnica National University of Science and Technology, 060042 Bucharest, Romania

**Keywords:** acute lymphoblastic leukemia, pediatric patients, ALL IC BFM, survival rates

## Abstract

*Background and Objectives*: Due to the progress made in all areas of research, pediatric patients diagnosed with acute lymphoblastic leukemia (ALL) now have an average overall survival rate of 90%. There are still discrepancies between high-income countries and limited-resource centers. The aim of this study was to analyze prognostic factors and outcome parameters in a 223-patient cohort from a single center in Romania, treated with two adapted BFM protocols. *Materials and Methods*: The patients diagnosed with ALL in our center were enrolled in this study from January 2016 to December 2022 and subsequently followed up until December 2024. The patients were treated first according to the ALL IC BFM 2009 protocol until June 2019 and afterwards with the ALL AIEOP BFM 2017 protocol starting with July 2019. The prognostic factors were analyzed in both subgroups and the outcomes were measured: event-free survival (EFS), overall survival (OS), cumulative incidence of relapse (CIR), relapse-free survival (RFS) and non-relapse mortality (NRM). *Results*: The comparison between the two subgroups revealed that every parameter improved over time: complete remission after induction (87.75% vs. 80.7%), early deaths (3.92% vs. 5.78%), deaths in remission (4.08% vs. 5.26%), 5-year EFS (73.79% vs. 70.22%), 5-year CIR (18.36% vs. 19.04%), 5-year RFS (81.76% vs. 80.97%), 5-year NRM (7.85% vs. 10.77%), and 5-year OS (88.18% vs. 82.54%). Whereas for the standard-risk group, events such as relapse or death were isolated, for intermediate-risk patients, the events were limited to a small number and did not significantly influence the overall results, and for high-risk children, the results improved significantly between the two subgroups. The worst outcomes were observed in patients with the BCR::ABL1 fusion gene, T-cell phenotype, and in teenagers, compared to the ETV6::RUNX1 fusion gene, B precursor ALL, and in smaller children, respectively. *Conclusions*: The 5-year OS increased in our center from 82.54% to almost 90%, with the most substantial finding being the survival rate for high-risk patients, now reaching up to 80%. The prognostic factors were age at diagnosis, genetic characteristics, and response to treatment, especially prednisone sensibility.

## 1. Introduction

Acute lymphoblastic leukemia (ALL) represents the most prevalent malignancy in the pediatric population and has been the subject of extensive clinical and translational research. Over recent decades, advances in treatment strategies have transformed ALL into a paradigmatic success in pediatric oncology, with overall survival rates now approaching 90% following the implementation of multi-agent chemotherapy regimens and the refinement of risk stratification methods [1,2,3]. The Berlin-Frankfurt-Münster (BFM) study group has played a central role in the development of standardized treatment protocols for ALL, which are widely used in various healthcare settings. Notably, the ALL IC-BFM subgroup was established to adapt these protocols for use in resource-limited contexts [4], and this therapeutic framework has been adopted in Romania during the past decade.

The national average incidence of ALL is 100 new cases/year, and the Fundeni Clinical Institute is responsible for diagnosing 40% of them, according to The Romanian Cancer Registry for Children with Oncological Diseases. Comparing 2010–2013 and 2014–2017 cohorts, the 2024 report showed an improvement in nationwide overall survival after 1, 3, and 5 years: 85%, 76%, and 74% versus 90%, 82%, and 80% [5].

The aim of this study was to analyze the survival of children with ALL, focusing on front-line therapy. The primary objective was to compare two therapeutic strategies based on BFM protocols by assessing the outcome parameters: event-free survival (EFS), overall survival (OS), cumulative incidence of relapse (CIR), relapse-free survival (RFS), and non-relapse mortality (NRM). The secondary objective was to evaluate prognostic factors and to identify those which have an impact on survival.

## 2. Materials and Methods

We performed a retrospective analysis for children diagnosed with ALL in the Fundeni Clinical Institute, between January 2016 and December 2022, with a follow-up period extending until December 2024. The inclusion criteria were (1) 0–18-year-old patients and (2) newly diagnosed patients with ALL. The exclusion criteria were (1) L3 leukemia and (2) mixed lineage phenotype leukemia. The discontinuation criteria were (1) patients lost to follow-up due to emigration to other countries at any point during first-line treatment, and (2) patients transferred to adult departments after turning 18.

The legal guardians signed informed consent forms for the diagnosis and treatment procedures, and for the participation in the medical scientific activity, respectively.

### 2.1. Specific Diagnostic and Monitoring Procedures

All the patients were diagnosed using bone marrow aspiration morphology and flowcytometry (FCM) results, conventional cytogenetic procedures with GTG banding, fluorescent-in situ-hybridization and molecular biology via polymerase-chain-reaction methods for the most common anomalies: BCR::ABL1, TCF3::PBX1, SIL::TAL1, KMT2A::AFF1, ETV6::RUNX1, FLT3-ITD duplication, and KMT2A::MLLT3.

We used “Genetic risk groups” to divide the patients into four categories:

1. Adverse prognosis = hypodiploidy/BCR::ABL1/KMT2A;
2. Favorable prognosis = ETV6::RUNX1/hyperdiploidy);
3. Other = identified mutations with no stratification impact;
4. None = unidentified mutations by classical methods, previously described.

MRD assessments were performed by FCM in all patients on days 15 (D15), 33 (D33, TP1), and 78 (D78, TP2), as recommended by the international protocols. According to the recommendations of the EuroFlow consortium, an eight-color panel was used, and a sensitivity level of 10^−4^ was achieved. Positive MRD was defined as the presence of at least 50 clustered events exhibiting lymphoid-scattering properties and leukemia-associated immunophenotypic features noted at diagnosis.

Prednisone response was assessed on day 8 using morphological analysis of peripheral blood smears, thus defining prednisone-good-responders (PGRs) and prednisone-poor-responders (PPRs). Complete remission (CR) by the end of induction (EOI) was established through morphology and FCM (MRD level of <0.05 × 10^−4^). Relapse was defined using classical parameters: reoccurrence of blasts in medullary or extramedullary sites. The early mortality rate was defined for the patients who died before EOI, and the mortality rate after achieving CR was applied to the children who died due to relapses or treatment-related causes (NRM). EFS was the time elapsed from diagnosis to an event, defined by relapse or death, while OS was the total period from diagnosis to death or the end of the study, 31 December 2024.

### 2.2. Treatment Protocols

The therapy strategy was based on BFM protocols, following standard arms, without randomizations, conveying a uniform management of our cohort. The patients were stratified into the standard-risk group (SRG), intermediate-risk group (IRG), and high-risk group (HRG), based on the international criteria (see below). BCR::ABL1 positive patients received tyrosine-kinase inhibitors, added to standard chemotherapy. There were a few adjustments to the protocol in all children, according to the local standard operational procedure:

- Intravenous dexamethasone used in induction, after a week of oral prednisone, to improve treatment adherence of small children by using intravenous administration instead of several tablets administered orally.
- No prophylactic use of irradiation therapy, due to logistical difficulties and no evidence-based studies showing radiotherapy benefits compared to chemotherapy.
- Monthly pulses with 5-days of dexamethasone and single vincristine dose for high-risk patients during maintenance, as recommended by several international studies [6,7].

The cohort was divided into two subgroups, namely T1-cohort, from January 2016 to June 2019, and T2-cohort, from July 2019 to December 2022.

(1) T1 patients were treated according to the ALL IC BFM 2009 protocol. The stratification criteria for T1: HRG had PPR or BCR::ABL1 or KMT2A::AFF1 or hypodiploidy ≤ 44 chromosomes or D15 FCM-MRD ≥ 10% or no CR by TP1; SRG had age > 1 and <6 years; and leukocyte count at diagnosis < 20 × 10^9^/L; and PGR and D15 FCM-MRD < 0.1% and CR on TP1; IRG were the remaining patients.
(2) For T2, the treatment plan was based on the ALL AIEOP BFM 2017 protocol: HRG had PPR only in T-cell ALL or BCR::ABL1 or KMT2A::AFF1 or hypodiploidy ≤ 44 chromosomes or D15 FCM-MRD ≥ 10% or TP1 FCM-MRD ≥ 5 × 10^−4^; SRG D15 FCM-MRD < 0.1%; and negative FCM-MRD on TP1; IRG were the remaining patients.

As far as the treatment differences are concerned, in T1, native *E. coli* asparaginase was used during induction, high-risk blocks, and reinduction, compared to T2 in which pegylated formulation was available upfront. Considering methotrexate dosage in consolidation for non-HRG B-cell ALL patients, in T1 the patients received 2 g/m^2^, in T2 the patients received 5 g/m^2^.

### 2.3. Statistical Analysis

Data analysis was conducted using Python version 3.12.0 with the following libraries employed: Pandas version 2.2.3 for data management and preprocessing, NumPy version 2.0.1 for numerical computations and data manipulation, SciPy version 1.13.1 for statistical analysis including normality assessments and hypothesis testing, Lifelines version 0.30.0 for survival analyses such as Kaplan–Meier estimations, log-rank testing and Cox proportional hazards modeling, Matplotlib version 3.9.4 for generating graphical representations, including survival curves and other visualizations, and Forest Plot for creating forest plots to visualize hazard ratios and confidence intervals. The categorical variables were expressed as absolute counts and percentages. Comparisons between groups were conducted using Pearson’s chi-square test or Fisher’s exact test, as appropriate. The normality of the distribution was assessed using the Shapiro–Wilk test and visualization of histograms. Normally distributed variables were reported as mean ± standard deviation, while non-normally distributed variables were reported as medians with interquartile ranges (quartile 1, quartile 3). The differences between two normally distributed groups were evaluated using the t-test, while the differences between two non-normally distributed groups were assessed with the Mann–Whitney–Wilcoxon test. Kaplan–Meier curves were generated and analyzed for OS, EFS, and RFS, and compared to the log-rank test. CIR and NRM were assessed using the Aalen–Johnson model, accounting for competing risks. A univariate survival analysis was conducted using a univariate Cox proportional hazards model. The variables with a *p*-value below 0.1 in the univariate Cox proportional hazards model were included in the multivariate Cox proportional hazards model. In the cases where both “Genetics and molecular biology” and “Genetic risk groups” met the *p*-value threshold, “Genetic risk groups” was prioritized to avoid potential convergence issues in the Cox analysis. Multicollinearity was assessed based on the variance inflation factor or on whether the variable was already included in another collected scoring system (the risk group variable was excluded from the multivariate analysis, as it encompasses other variables that were individually included in the model). In instances where no events occurred in one of the selected groups, rendering the Cox proportional hazards model unsuitable, the log-rank test was used to calculate a *p*-value. The statistical significance was defined as a *p*-value lower than 0.05.

## 3. Results

A total number of 223 patients met the inclusion/exclusion/discontinuation criteria (the medium attrition rate during first-line therapy was approximately 17% annually). The rate of complete follow-up was 97.31%. Table 1 summarizes the general characteristics of the entire cohort, specifically the demographic and diagnostic parameters at onset, but also the characteristics of the two subgroups, represented by T1 (121 patients) and T2 (102 patients).

Overall, there was a slight male predominance in both groups, with 58.68% in T1 and 59.80% in T2. Most patients were between 1 and 10 years old, representing 68.60% of the first treatment group and 67.65% of the second. As expected, the predominant immunophenotype was B-cell lineage, observed in 87.60% of patients in T1 and 88.24% in T2.

The T1 cohort comprised 15 patients (12.39%) SRG, 66 patients (54.54%) IRG, 36 patients (29.75%) HRG, and 4 patients (3.3%) not included in any category, due to death EOI and no prior HRG characteristics. In T2 the distribution was 21 patients (20.58%) SRG, 45 patients (44.11%) IRG, 33 patients (32.35%) HRG, and 3 patients (2.94%) not included in any category.

The rate of CR at EOI was 80.7% in T1, and 87.75% in T2; death-before-EOI rate was 5.78% in T1 and 3.92% in T2; death-in-CR rate was 5.26% in T1 and 4.08% in T2; the CIR was 19.04% in T1 and 18.36% in T2. Regarding the mortality causes for the patients achieving CR, mention should be made of severe sepsis and post-hematopoietic stem cell transplantation (HSCT) toxicities.

Out of the 223 patients, 9 of them underwent HSCT in the first CR according to the BFM indications, respectively, 2 children from T1 (1 transitioned to adult care and was lost to follow-up and 1 died due to post-HSCT toxicities), and 7 children from T2 (6/7 remained in first CR, only 1/7 experienced a post-HSCT relapse and eventually succumbed to the disease). A total of 13 patients received HSCT in ≥2nd CR, respectively, 9 from T1 (4/9 are in CR, 2/9 died due to post-HSCT toxicities, and 3/9 died due to disease progression) and 4 from T2 (3/4 are in CR, only 1 died because of severe toxicity).

It should be noted that the median follow-up for T1 patients was 88.83 months and 46.72 months for the T2 sub cohort, indicating a longer observation period and more mature survival data for the former subgroup. To address this aspect, we further conducted an additional sensitivity analysis, in which we truncated the follow-up duration of both subgroups, to ensure unbiased comparison. The results of this analysis were consistent with those of the original analysis, while small sporadic differences were observed in the number of events between groups, these differences were not substantial enough to alter the overall trends in survival curves. Additional information is available in the Supplementary Materials.

**Figure 1:**
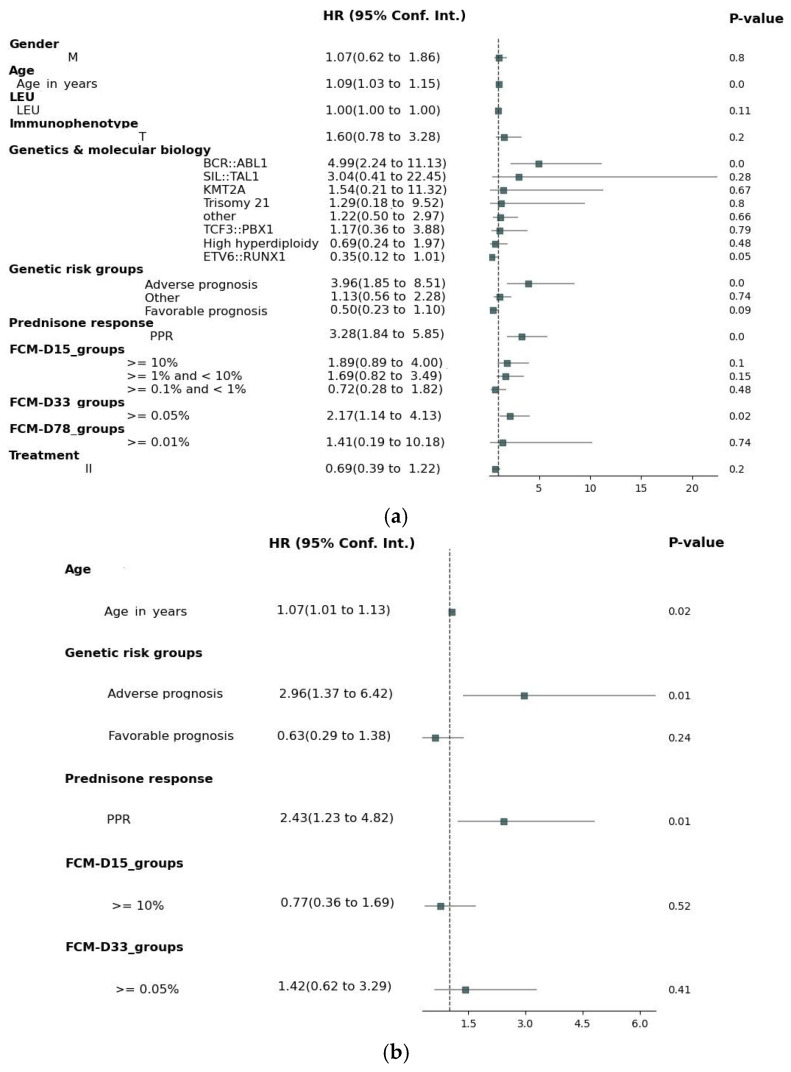
Cox analysis on EFS. (**a**) Univariate Cox analysis; (**b**) multivariate Cox analysis. Adverse prognosis = hypodiploidy/BCR::ABL1/KMT2A; favorable prognosis = ETV6::RUNX1/hyperdiploidy; other = identified mutations with no stratification impact.

Figure 1a presents the results of the univariate Cox regression analysis for EFS. Several factors demonstrated significant associations with EFS: age, genetic factors, prednisone response, FCM MRD on D15 and D33. For the subsequent multivariate analysis, all the variables with a univariate *p*-value ≤ 0.1 were selected. In the cases where the parameters from both the “Genetics and molecular biology” and “Genetic risk groups” classification met the *p*-value threshold, the latter was prioritized to avoid potential convergence issues in the Cox analysis.

Figure 1b presents the results of the multivariate Cox proportional hazards regression analysis for EFS. While the MRD levels on D15 (HR = 0.77, 95% CI: 0.36–1.69, *p* = 0.52) and D33 (HR = 1.42, 95% CI: 0.62–3.29, *p* = 0.40) did not maintain statistical significance, adverse genetic prognosis (HR = 2.96, 95% CI: 1.37–6.42, *p* = 0.005), poor prednisone response (HR = 2.43, 95% CI: 1.23–4.82, *p* = 0.01), and age (HR = 1.07, 95% CI: 1.01–1.13, *p* = 0.02) retained their significant association with EFS.

**Figure 2:**
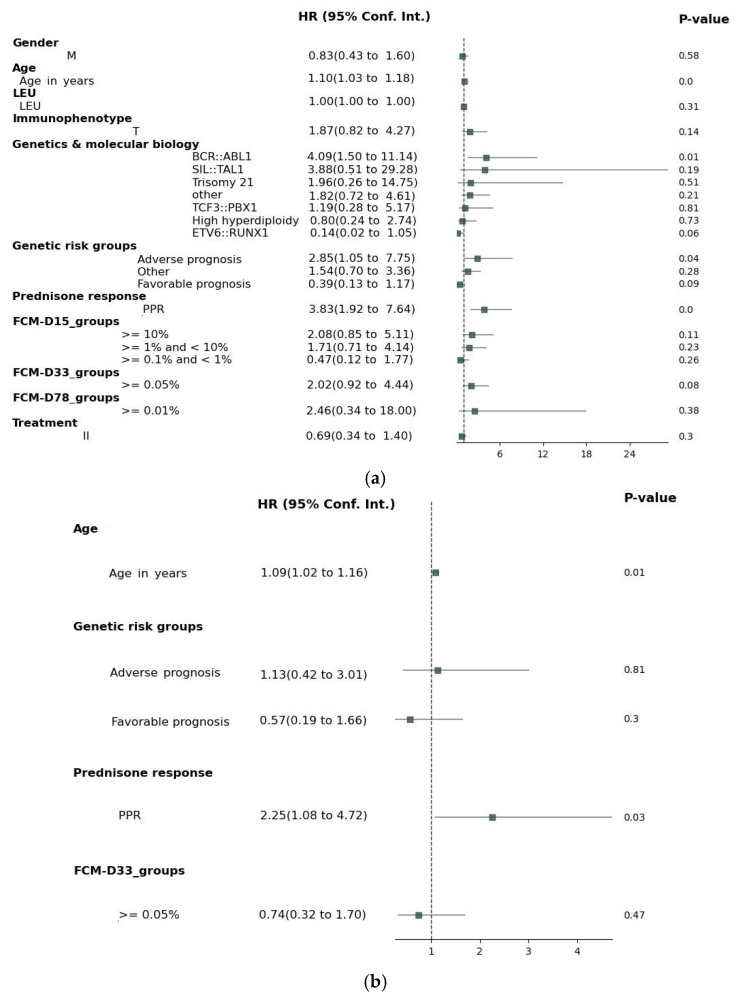
Cox analysis on OS. (**a**) Univariate Cox analysis; (**b**) multivariate Cox analysis. Adverse prognosis = hypodiploidy/BCR::ABL1/KMT2A; favorable prognosis = ETV6::RUNX1/hyperdiploidy; other = identified mutations with no stratification impact.

In the univariate analysis of OS for the entire cohort (Figure 2a), several factors were identified as potential prognostic indicators. Increasing age (HR 1.10, 95% CI 1.03–1.18, *p* = 0.003, Wald) and prednisone poor response (HR 3.86, 95% CI 1.93–7.70, *p* < 0.001, Wald) were significantly associated with an increased risk of death. The presence of BCR::ABL1 (HR 4.09, 95% CI 1.50–11.14, *p* = 0.006, Wald) was associated with significantly worse OS. In the “Genetic risk groups”, the adverse prognosis category (HR 2.85, 95% CI 1.05–7.75, *p* = 0.040, Wald) was associated with a higher risk of mortality, while a favorable prognosis was borderline significant (HR 0.39, 95% CI 0.13–1.17, *p* = 0.094, Wald), and was further included in the multivariate analysis. The MRD assessment using FCM ≥ 0.05% on Day 33 was significantly associated with a worse prognosis (HR 2.02, 95% CI 0.92–4.44, *p* = 0.081, Wald). However, the MRD levels on D15 and D78, as well as other genetic abnormalities, immunophenotype, treatment, and gender, did not show significant associations with OS. In the multivariate Cox regression analysis of OS (Figure 2b), several factors were found to significantly influence survival outcomes. Age (HR 1.09, 95% CI 1.02–1.16, *p* = 0.014) was significantly associated with an increased risk of mortality, with each year increase in age corresponding to a higher risk of death. Poor prednisone response (PPR; HR 2.25, 95% CI 1.08–4.72, *p* = 0.031) was also significantly associated with worse survival. However, FCM-MRD on Day 33 did not maintain statistical significance in the multivariate model (HR 0.74, 95% CI 0.32–1.70, *p* = 0.473). Genetic factors were not statistically significant for OS in this multivariate analysis (HR 0.81, 95% CI 0.42–3.01, *p* = 0.81 for “adverse prognosis” and HR 0.57, 95% CI 0.19–1.66, *p* = 0.3 for “favorable prognosis”).

**Figure 3:**
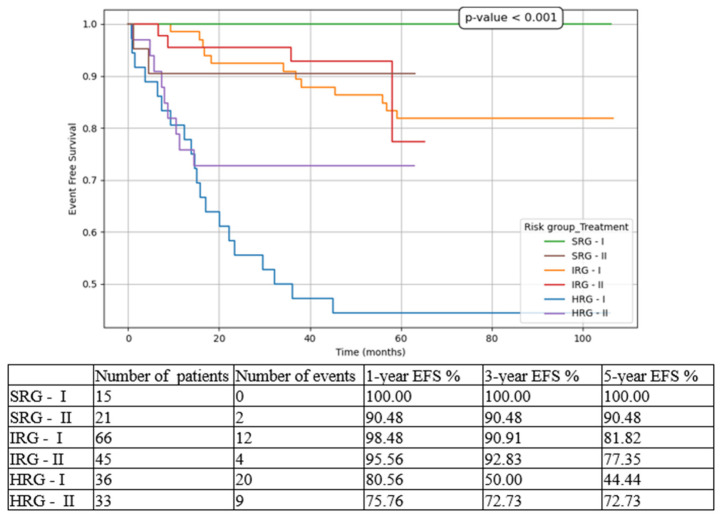
Event-free survival (EFS) based on risk groups and treatment options. SRG-I = SRG patients in T1, SRG-II = SRG patients in T2, IRG-I = IRG patients in T1, IRG-II = IRG patients in T2, HRG-I = HRG patients in T1, HRG-II = HRG patients in T2.

**Figure 4:**
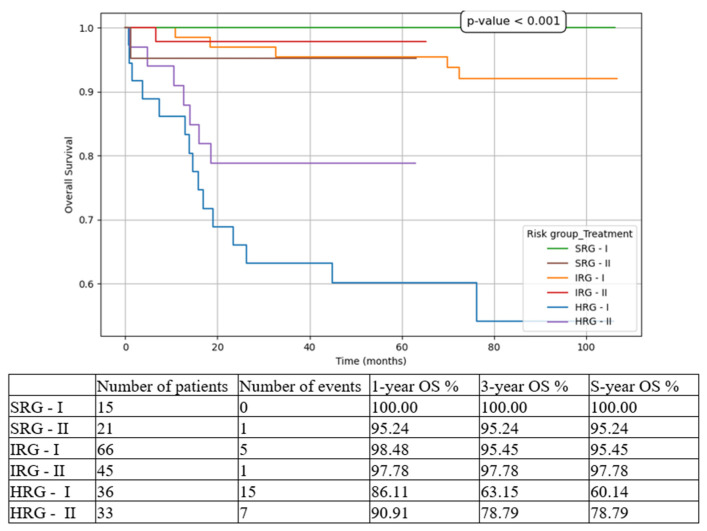
Overall survival (OS) based on risk groups and treatment options. SRG-I = SRG patients in T1, SRG-II = SRG patients in T2, IRG-I = IRG patients in T1, IRG-II = IRG patients in T2, HRG-I = HRG patients in T1, HRG-II = HRG patients in T2.

**Figure 5:**
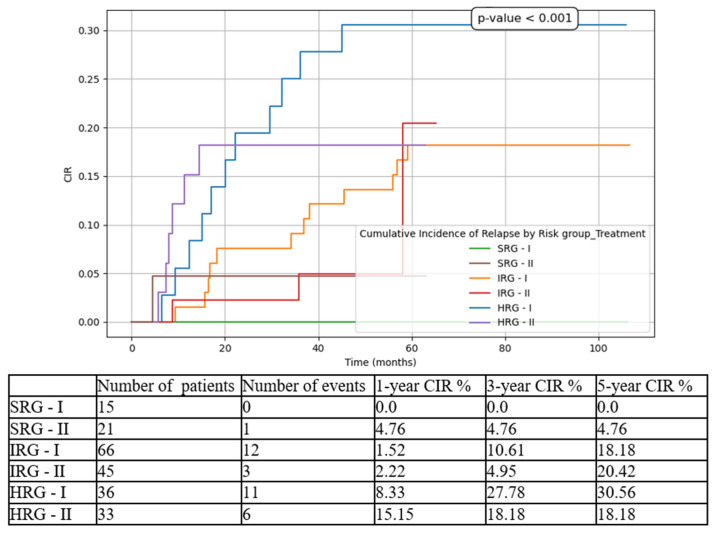
Cumulative incidence of relapse (CIR) based on risk groups and treatment options. SRG-I = SRG patients in T1, SRG-II = SRG patients in T2, IRG-I = IRG patients in T1, IRG-II = IRG patients in T2, HRG-I = HRG patients in T1, HRG-II = HRG patients in T2.

**Figure 6:**
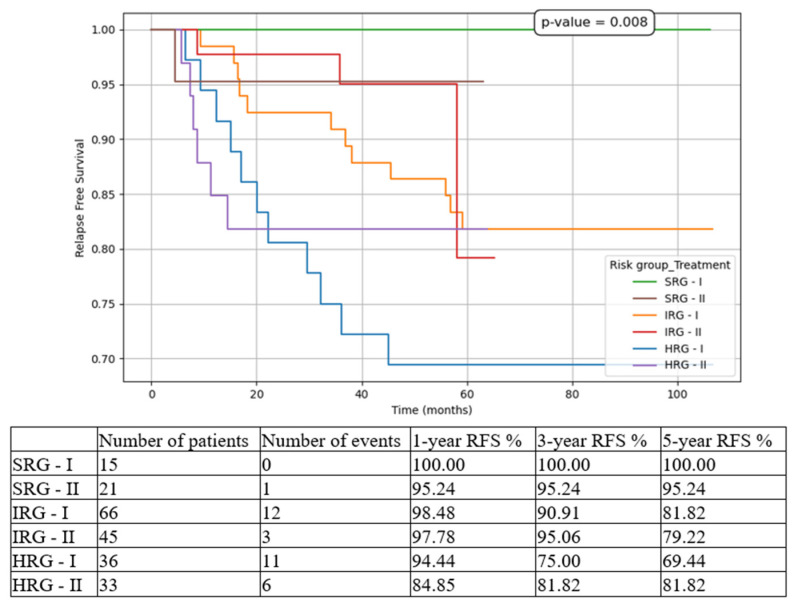
Relapse-free survival (RFS) based on risk groups and treatment options. SRG-I = SRG patients in T1, SRG-II = SRG patients in T2, IRG-I = IRG patients in T1, IRG-II = IRG patients in T2, HRG-I = HRG patients in T1, HRG-II = HRG patients in T2.

**Figure 7:**
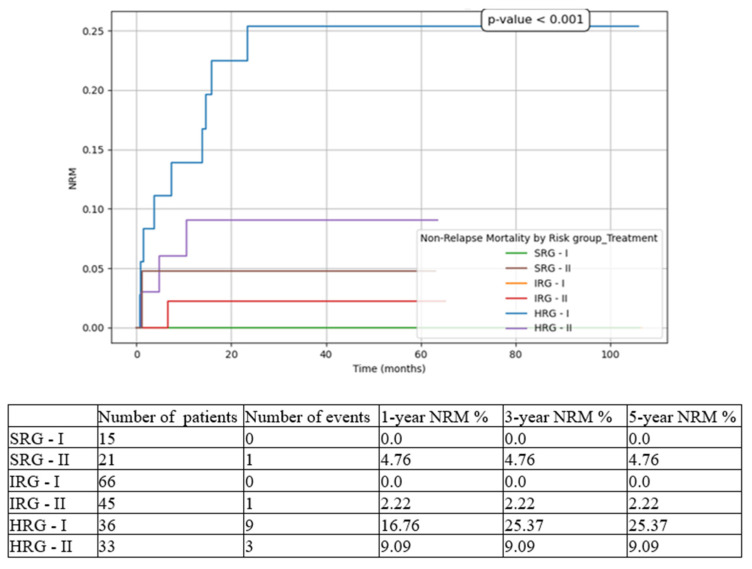
Non-relapse mortality (NRM) based on risk groups and treatment options. SRG-I = SRG patients in T1, SRG-II = SRG patients in T2, IRG-I = IRG patients in T1, IRG-II = IRG patients in T2, HRG-I = HRG patients in T1, HRG-II = HRG patients in T2.

We compared the three risk-group patients between the two cohorts (T1 vs. T2). The most statistically significant results were for HRG patients, with improvements in all contexts:

- 5-year EFS from 44.44% in T1 to 72.73% in T2 (Figure 3).
- 5-year OS from 60.14% in T1 to 78.79% in T2 (Figure 4).
- 5-year CIR from 30.56% in T1 to 18.18% in T2 (Figure 5).
- 5-year RFS from 69.44% in T1 to 81.82% in T2 (Figure 6).
- 5-year NRM from 25.37% in T1 to 9.09% in T2 (Figure 7).

## 4. Discussion

The examination of the entire cohort reveals that there was a slight male predominance (59.2%), with most patients being between the ages of 1 and 10 (68.16%) and only two patients bellow the age of one; the main immunophenotype was B-cell lineage (87.89%). These findings are consistent with the international papers on large cohorts [2,8,9]. The most frequent genetic feature was ETV6::RUNX1 (18.83%), followed by hyperdiploidy (10.31%) and TCF3::PBX1 (5.38%); less common mutations were BCR::ABL1 (4.47%), KMT2A rearrangements (1.34%) and SIL::TAL1 (0.89%); the incidence of Down syndrome was 1.34% and there were no patients with hypodiploidy. Whereas in almost 48% of children no genetic abnormalities were found, either due to a normal karyotype, or to the absence of metaphases, in the remaining 9.41%, the tests uncovered several cytogenetic lesions, but they were not important in terms of risk group stratification.

EFS was 87.9% (CI 82.33–91.15%) at 1 year, 79.6% (CI 73.69–84.38%) at 3 years, and 73.7% (CI 66.90–79.37%) at 5 years. OS was 91.9% (CI 86.94–94.47%) at 1 year, 85.5% (CI 80.14–89.53%) at 3 years, and 84.9% (CI 79.46–89.06%) at 5 years. The resulting values for survival presented in this study were higher than those reported from middle-income countries on large cohorts: 65% EFS and 71.9% OS at 5 years from the Brazilian groups [10], 3-year EFS of 71% and OS of 79.6% in other centers from Latin America [11], 5-year estimated survival rate in Armenia of 75% [12], or 3-year EFS of 52% and OS of 58% in India [9]. Nevertheless, high-income countries exceed the values presented in this report, with a 5-year OS of 89% in England, 91% in the Netherlands, and 94% in the United States of America [10].

For the univariate Cox regression analysis, we evaluated general patient characteristics and disease biological features, such as gender, age, immunophenotype, specific genetic characteristics, further categorized according to a known genetic prognosis impact, and treatment response, represented by prednisone sensibility and FCM-MRD on D15, D33, and D78. The risk groups were not included in the uni- or multivariate analyses due to the fact that they were assigned according to other included factors. The results of the univariate Cox regression analysis for EFS showed significant associations with the patient’s genetic profile at diagnosis, prednisone response, D15, and D33 FCM-MRD (D78 FCM-MRD did not show statistical significance, but there was a very small number of patients with MRD ≥ 0.01% in TP2). In the multivariate Cox, only age, poor genetic traits, and prednisone response retained noteworthy correlations. In the univariate analysis of OS, several factors were identified as potential prognostic indicators: age, genetic aspects at onset of the disease, prednisone response, and D33 FCM-MRD. Nevertheless, in the multivariate Cox regression, the only factors which maintained statistical significance in our cohort were age at diagnosis and blast clearance one week after oral prednisone. Regarding the genetic abnormalities discovered at diagnosis, mention should be made that ETV6::RUNX1 and BCR::ABL1 fusion genes were, respectively, at the opposite spectrum for influencing the children’s outcomes, as specified in other studies [13,14,15].

Our results are congruent with the international reports [8,9,16]. The fact that age and prednisone response are still important independent predictors for EFS and OS in children with ALL raises the question whether their exclusion from the latest stratification criteria is warranted. It remains unclear why the MRD assessment did not preserve an independent statistical significance for our cohort, the hypothesis being that the treatment response is already influenced by the patient’s characteristics, such as age and chromosomal and molecular abnormalities, as mentioned in the previous papers [17,18,19,20].

Even though the immunophenotype was not a statistical predictor for outcome in our cohort, it is worth mentioning that T-ALL patients were only 12%. Even though the *p*-value was consistently higher than 0.05, all prognostic parameters after 5 years (EFS, OS, NRM, CIR, RFS) were decreased in T-ALL children compared to B-ALL patients: 64.51% vs. 75.08, 77.29% vs. 85.98%, 14.98% vs. 8.68%, 20.68% vs. 16.24%, and 79.86% vs. 83.78%, respectively. The reasons for these findings are probably the undefined genetic stratification criteria and the higher prevalence of T-ALL in adolescents [21], who are prone to worse outcomes due to a different leukemic pathology, poor tolerance of chemotherapy, and even suboptimal adherence to therapy [22,23].

The 223-patient cohort was divided into two subgroups based on the date of diagnosis and the treatment protocol used, i.e., 121 patients were included in the first group (T1) and 102 in the second group (T2). The quality assessment parameters are presented in Table 2. As far as the differences in risk group stratifications are concerned, they are easily explained by the changes in criteria: prednisone response was maintained only for T-ALL patients, and the number of leukocytes and age at diagnosis were removed from the classification in the ALL AIEOP BFM 2017 protocol, compared to the ALL IC BFM 2009 strategy.

Taking into account the official results of the ALL IC BFM 2009 protocol published in 2023 [4], we were able to compare the outcomes of our 121 children from T1 group with the 6187 patients from the aforementioned study. It should be emphasized that our patients followed the standard arm of the protocol, without randomization at any point during the treatment. Looking at the 5-year global results, the OS is almost identical (82.54% vs. 82.6%), but the EFS is better in the international paper (75.2% vs. 70.22%). An explanation for the 5% difference could be the higher incidence in our cohort both for children 10–18-years-old (31.4% vs. 23.9%), and for the patients with BCR::ABL1 (5.79% vs. 2%), both categories being known for a higher incidence of relapse and worse outcome on the overall.

The percentages for the three risk groups were slightly different in our analysis (SRG 12.39% vs. 10%, IRG 54.54% vs. 66.5%, and HRG 29.75% vs. 23.5%). Insofar as the outcomes are concerned, for SRG and IRG, there were no discrepancies between the two cohorts, in terms of EFS and OS. However, the results were not encouraging in our cohort for HRG patients, with an EFS of 44.44% vs. 60.8% and an OS of 60.14% vs. 68.4%. The other parameters did not differ significantly (survival in B-ALL vs. T-ALL, death-in-CR and relapse rates), except for early deaths in induction (5.78% vs. 1.7%).

We proceeded to compare the results from two periods of time: 2016–2019 and 2019–2022. As presented in Table 2, although there was not a statistically significant difference (EFS *p* = 0.24, CIR *p* = 0.24, OS *p* = 0.335, NRM *p* = 0.483, RFS *p* = 0.321), the improvement is obvious if we consider the aforementioned values. Moreover, the previous 5-year OS for Romanian patients was 74% for the 2010–2013 cohort, and 80% for the 2014–2017 cohort [5].

While for the SRG patient events such as relapse or death were isolated, and for the IRG patients they were limited to a small number and did not significantly influence the overall outcomes, for the HRG children the results drastically changed between the two subgroups. There was an important increase in 5-year EFS from 44.44% to 72.73% (*p* < 0.001), in 5-year OS from 60.14% to 78.79% (*p* < 0.001), and in 5-year RFS from 69.44% to 81.82% (*p* = 0.008), on the one hand, and a decrease in 5-year CIR from 30.56% to 18.18% (*p* < 0.001), and in 5-year NRM from 25.37% to 9.09% (*p* < 0.001), on the other hand. Similarly, other groups have reported increasing outcomes over the last decade [6,24].

It should be emphasized that CIR was higher for IRG, compared to HRG, both in the T1 and the T2 cohorts. The explanation might reside in the fact that the IRG patients are a heterogeneous group, who might benefit from a subclassification based on genetic characteristics and/or D15 FCM-MRD (for example, 0.1–1% intermediate-low and 1–10% intermediate-high). More to the point, using triple intrathecal chemotherapy during the consolidation phase might decrease the incidence of CNS relapses in IRG patients, but these proposals need to be assessed in large multicentric studies.

Most treatment protocols were developed and delivered in high-income countries; therefore, it is necessary to have adjusted regimens, based on local means in middle- or low-income countries using standardized regimens, such as ALL IC BFM [25]. As stated by a Polish group, most countries with high health expenditure report lower death rates in ALL children, due to any cause, compared to less experienced teams or with fewer resources [26]. It is similarly important to understand that treatment efficacy is not only determined by EFS and OS, but also by frequency and severity of toxicities. Hence, future de-escalation strategies for the SRG patients should be the focus, without adversely affecting the cure rate [17,27].

There were limitations in this paper: the retrospective study, the difference in median follow-up between the two subgroups, and the single center cohort. Even though a retrospective approach can lead to a biased analysis, we created a uniform database: (1) by enrolling all consecutively diagnosed children who met the inclusion/exclusion criteria, (2) by retrieving the same data about the participants, (3) by eliminating upfront information that was not available for all patients (PCR-MRD, central nervous system status), and (4) by excluding the parameters which were influenced on admission in the Fundeni Clinical Institute (hemoglobin or platelet levels due to prior transfusions, white blood count impacted by hyperhydration or lack thereof). Insofar as the second limitation is concerned, i.e., the discrepancy between the median follow-up after 88 months for the T1 patients and 44 months for the T2 patients, we do not consider it to be an influential limitation, since most relapses and deaths occur prior to the 3-year mark after diagnosis, as shown in Section 3 of this paper. It is important to highlight that the Kaplan–Meier method inherently accounts for censoring, which helps mitigate this bias. Events and censoring are both incorporated into the calculation of survival probabilities at each time point, allowing for an appropriate estimate of survival even in the presence of differing follow-up durations—as long as the assumption of non-informative censoring holds. The third limitation we identified is the single center experience report, but according to the National Cancer Registry report [3], the Fundeni Clinical Institute diagnoses and treats 40% of ALL cases; therefore, we consider this analysis to be significant.

## 5. Conclusions

To conclude, we have shown increasing survival rates in children diagnosed with ALL and treated in the Fundeni Clinical Institute, with a global 5-year OS rising from 82.54% in 2016–2019 to almost 90% in 2019–2022, presently close to the figures published by international consortia. The most significant results were for HRG, which demonstrated an increase in OS up to a value close to 80%. In our cohort, independent inferior prognostic factors were older age and poor prednisone response, while the univariate analysis also listed unfavorable genetic characteristics and MRD assessments. Additional optimization of personalized therapy is required for further improving the outcome and diminishing disease- and treatment-related mortality.

## Figures and Tables

**Table 1:** Overview of diagnostic characteristics for cohort and for two subgroups.

		Total (*n* = 223)	T1 (*n* = 121)	T2 (*n* = 102)	*p*-Value
Gender	Female	91 (40.8%)	50 (41.32%)	41 (40.20%)	0.892
	Male	132 (59.2%)	71 (58.68%)	61 (59.80%)	
Age groups	<1 y	2 (0.90%)	0 (0.00%)	2 (1.96%)	0.301
	≥1 y and <10 y	152 (68.16%)	83 (68.60%)	69 (67.65%)	
	≥10 y	69 (30.94%)	38 (31.40%)	31 (30.39%)	
Leukocyte groups	<50 × 10^9^/L	175 (78.47%)	97 (80.17%)	78 (76.47%)	0.518
	≥50 × 10^9^/L	48 (21.53%)	24 (19.83%)	24 (23.53%)	
Morphology	L1	213 (95.51%)	116 (95.87%)	98 (96.08%)	1
	L2	9 (4.03%)	5 (4.13%)	4 (3.92%)	
Immunophenotype	B	196 (87.89%)	106 (87.60%)	90 (88.24%)	1
	T	27 (12.11%)	15 (12.40%)	12 (11.76%)	
Genetics and molecular biology	Hyperdiploidy	23 (10.31%)	11 (9.09%)	12 (11.76%)	0.305
	Trisomy 21	3 (1.34%)	3 (2.48%)	0 (0.00%)	
	KMT2A	3 (1.34%)	1 (0.83%)	2 (1.96%)	
	TCF3::PBX1	12 (5.38%)	4 (3.31%)	8 (7.84%)	
	ETV6::RUNX1	42 (18.83%)	23 (19.01%)	19 (18.63%)	
	SIL::TAL1	2 (0.89%)	0 (0.00%)	2 (1.96%)	
	BCR::ABL1	10 (4.47%)	7 (5.79%)	3 (2.94%)	
	Other	21 (9.41%)	13 (10.74%)	8 (7.84%)	
	None	107 (47.98%)	59 (48.76%)	48 (47.05%)	
Genetic risk groups	Adverse prognosis	13 (5.82%)	8 (6.61%)	5 (4.90%)	0.895
	Favorable prognosis	61 (27.35%)	31 (25.62%)	30 (29.41%)	
	Other	42 (18.83%)	23 (19.01%)	19 (18.62%)	
	None	107 (47.98%)	59 (48.76%)	48 (47.06%)	

*n* = number; y = years; adverse prognosis = hypodiploidy/BCR::ABL1/KMT2A; favorable prognosis = ETV6::RUNX1/hyperdiploidy; other = identified mutations with no stratification impact; none = unidentified mutations by classical methods.

**Table 2:** Overview of quality assessment in two subgroups.

	T1	T2
Risk stratification	SRG 12.39%	SRG 20.58%
	IRG 54.54%	IRG 44.11%
	HRG 29.75%	HRG 32.35%
FCM-MRD < 0.05 × 10^−4^ rate at EOI	80.7%	87.75%
5-year EFS	70.22%	73.79%
5-year CIR	19.04%	18.36%
5-year OS	82.54%	88.18%
Death-before-EOI rate	5.78%	3.92%
Death-in-CR rate	5.26%	4.08%
5-year NRM	10.77%	7.85%
5-year RFS	80.97%	81.76%

## Data Availability

Data is unavailable due to privacy. The raw data supporting the conclusions of this article will be made available by the authors on request.

## Supplementary Materials

The following supporting information can be downloaded at: https://www.mdpi.com/article/10.3390/medicina61071129/s1. Figures S1–S5: Overall survival (OS), event-free survival (EFS), relapse-free survival (RFS), non-relapse mortality (NRM), and cumulative incidence of relapse (CIR) based on risk group and treatment options; Figure S6: Forest plot illustrating Cox regression analysis on OS. (A) Univariate Cox regression analysis. Variables with *p*-values < 0.1 were considered for multivariate analysis. (B) Multivariate Cox regression analysis; Figure S7: Forest plot illustrating Cox regression analysis on EFS. (A) Univariate Cox regression analysis. Variables with *p*-values < 0.1 were considered for multivariate analysis. (B) Multivariate Cox regression analysis.

## Abbreviations

The following abbreviations are used in this manuscript:

ALL	Acute lymphoblastic leukemia
BFM	Berlin-Frankfurt-Münster
CR	Complete remission
CI	Confidence interval
CIR	Cumulative incidence of relapse
D15	Day 15
D33, TP1	Day 33
D78, TP2	Day 78
EOI	End of induction
EFS	Event-free survival
FCM	Flowcytometry
HR	Hazard ratio
HSCT	Hematopoietic stem cell transplant
HRG	High-risk group
IRG	Intermediate-risk group
MRD	Measurable residual disease
NRM	Non-relapse mortality
OS	Overall survival
PGR	Prednisone good response
PPR	Prednisone poor response
RFS	Relapse-free survival
SRG	Standard-risk group
