# Supplementary material for: Changes in the Outcome of Pediatric Patients with Acute Lymphoblastic Leukemia—Single Center, Real-Life Experience
Source: Medicina (Kaunas). 2025 Jun 23;61(7):1129. doi: 10.3390/medicina61071129 (PMC12300199; doi:10.3390/medicina61071129)
Supplement: Supplementary file 1 [file medicina-61-01129-s001.zip › medicina-3642810-supplementary.pdf]

We conducted an additional sensitivity analysis in which we truncated the follow-up duration of both subgroups to 46 months, corresponding to the minimum median follow-up between the two subgroups, to ensure unbiased comparison. This allowed us to standardize the follow-up window across both treatment arms and eliminate the potential influence of longer follow-up in the T1 group.

The results of this analysis were consistent with those of the original analysis: while small, sporadic differences were observed in the number of events between groups at various time points, these differences were not substantial enough to alter the estimated 1-year and 3-year outcome rates (5-year estimates are not available in this analysis due to the shortened follow-up window). Importantly, the statistical comparison remained robust, and the overall trends in survival curves were preserved (see Figures S1–S5).

Regarding the Cox regression analyses, while we observed minor differences in hazard ratios and p-values between the original and sensitivity analyses, the overall trends remained consistent, as illustrated in the forest plots for both univariate and multivariate Cox analyses of overall survival (OS) and event-free survival (EFS).

For OS, the univariate analysis conducted in the sensitivity analysis confirmed the same statistically significant associations as those identified in the original analysis—specifically, age, prednisone response, and FCM-D33 ≥0.05%. A difference was observed in the genetic risk groups: only the adverse prognosis subgroup reached a p-value <0.1 and was subsequently included in the multivariate analysis. In contrast, both favorable and adverse prognosis subgroups met this threshold in the original analysis, although neither retained statistical significance in the multivariate model. Additionally, FCM-D15 ≥10% met the inclusion criterion for multivariate analysis in the sensitivity analysis (HR 2.60, 95% CI 0.97–6.92, p = 0.06), demonstrating a slightly stronger association compared to the original analysis (HR 2.08, 95% CI 0.85–5.11, p = 0.11). In the multivariate Cox regression for OS, age and prednisone response remained statistically significant predictors, consistent with the findings of the original analysis.

In the case of EFS, the sensitivity analysis identified the same variables with p-values <0.1 in the univariate model as in the original analysis—namely, age, genetic risk groups (adverse and favorable prognosis), prednisone response, and FCM-D33 ≥0.05%. Additionally, FCM-D15 ≥10% once again approached the threshold for inclusion (HR 1.97, 95% CI 0.91–4.24, p = 0.08), showing a slightly stronger association than in the original analysis (HR 1.89, 95% CI 0.89–4.00, p = 0.10). In the multivariate Cox regression, age and prednisone response remained statistically significant predictors. FCM-D15 ≥10% nearly approached statistical significance (p = 0.06), while the adverse prognosis genetic risk group no longer maintained significance (HR 1.71, 95% CI 0.79–3.73, p = 0.18), in contrast to the original model where it had met the inclusion threshold.

Figures S1–S5. Overall survival (OS), event free survival (EFS), relapse-free survival (RFS), non-relapse mortality (NRM) and cumulative incidence of relapse (CIR) based on risk group and treatment options

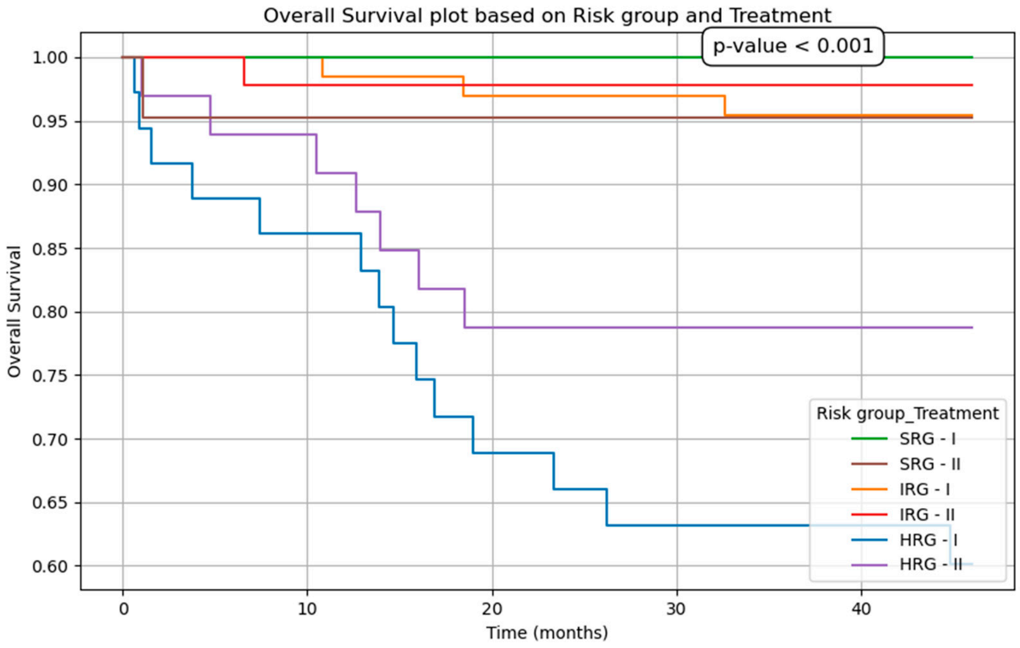

	Number of patients	Number of events	1-year Overall Survival %	3-year Overall Survival %
SRG - I	15	0	100.00	100.00
SRG - II	21	1	95.24	95.24
IRG - I	66	3	98.48	95.45
IRG - II	45	1	97.78	97.78
HRG - I	36	14	86.11	63.15
HRG - II	33	7	90.91	78.79

Figure S1

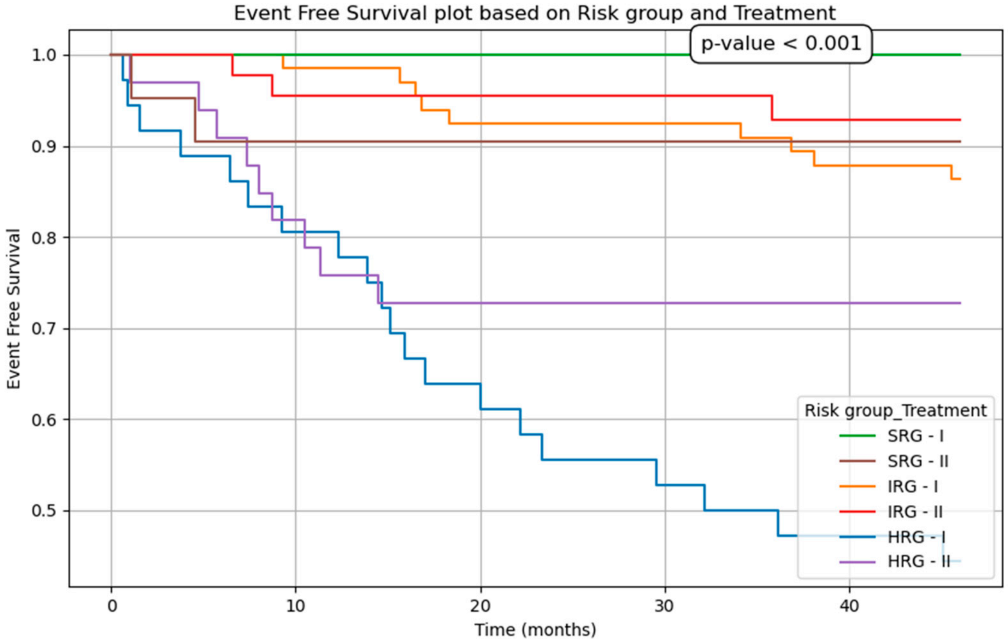

	Number of patients	Number of events	1-year EFS %	3-year EFS %
SRG - I	15	0	100.00	100.00
SRG - II	21	2	90.48	90.48
IRG - I	66	9	98.48	90.91
IRG - II	45	3	95.56	92.83
HRG - I	36	20	80.56	50.00
HRG - II	33	9	75.76	72.73

Figure S2

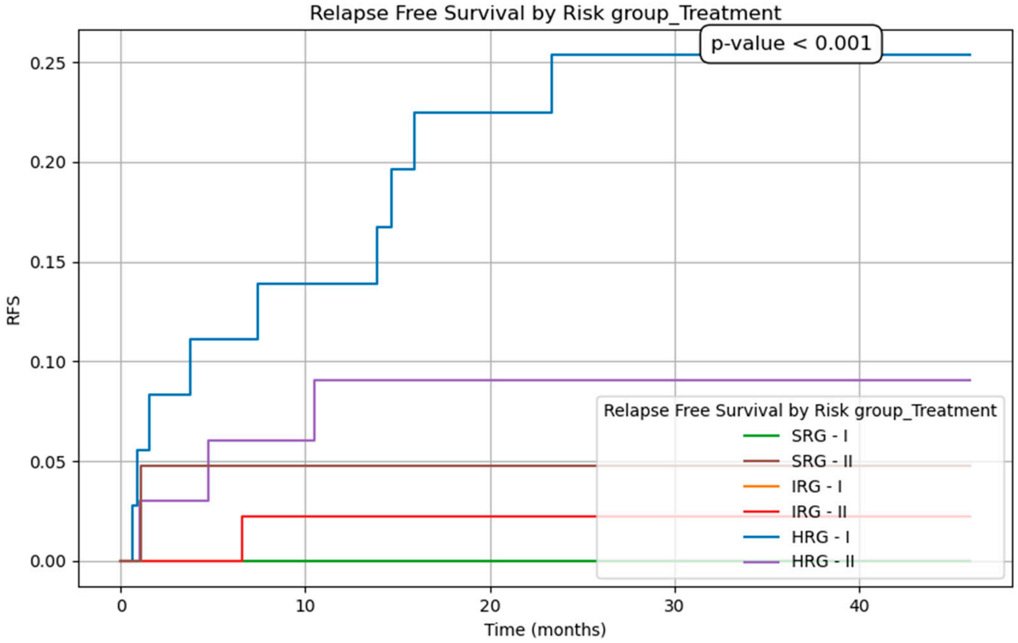

	Number of patients	Number of events	1-year Relapse Free Survival %	3-year Relapse Free Survival %
SRG - I	15	0	100.00	100.00
SRG - II	21	1	95.24	95.24
IRG - I	66	9	98.48	90.91
IRG - II	45	2	97.78	95.06
HRG - I	36	11	94.44	75.00
HRG - II	33	6	84.85	81.82

Figure S3

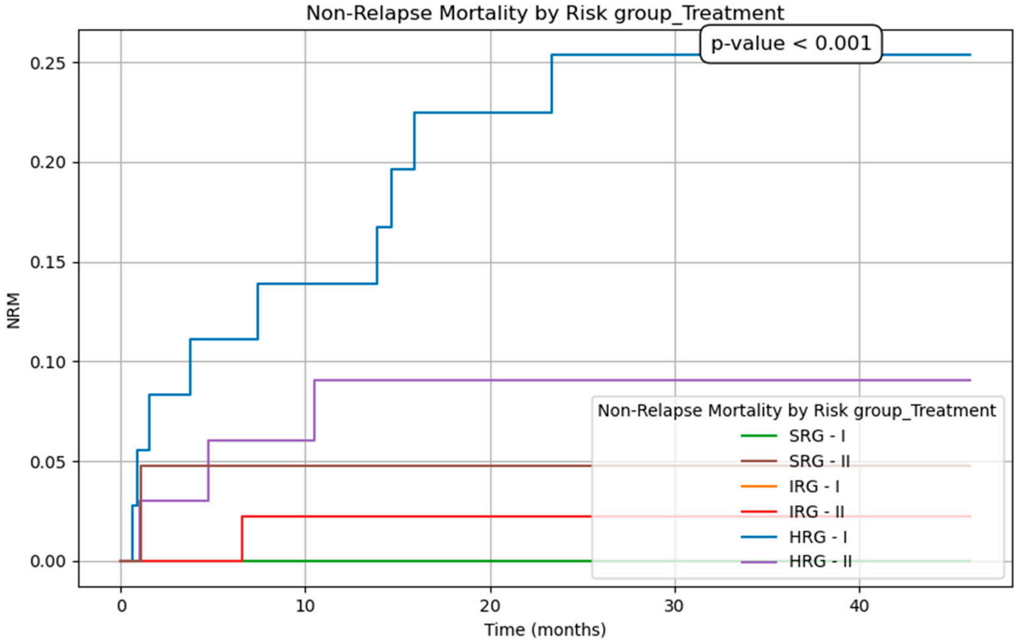

	Number of patients	Number of events	1-year NRM %	3-year NRM %
SRG - I	15	0	0.0	0.0
SRG - II	21	1	4.76	4.76
IRG - I	66	0	0.0	0.0
IRG - II	45	1	2.22	2.22
HRG - I	36	9	16.76	25.37
HRG - II	33	3	9.09	9.09

Figure S4

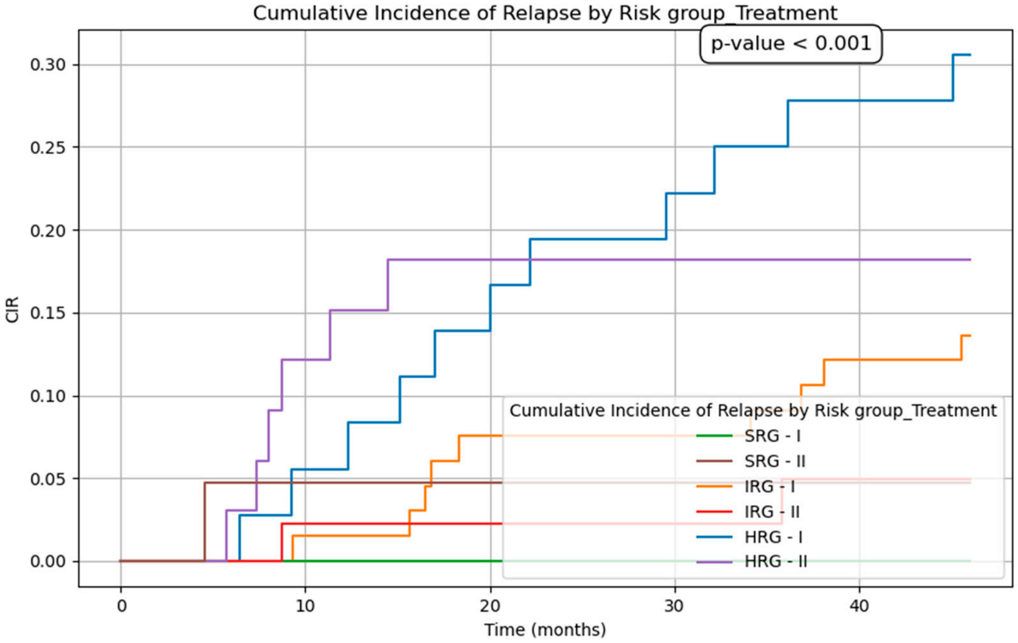

	Number of patients	Number of events	1-year CIR %	3-year CIR %
SRG - I	15	0	0.0	0.0
SRG - II	21	1	4.76	4.76
IRG - I	66	9	1.52	10.61
IRG - II	45	2	2.22	4.95
HRG - I	36	11	8.33	27.78
HRG - II	33	6	15.15	18.18

Figure S5

Figure S6. Forest plot illustrating Cox regression analysis on OS.

(A) Univariate Cox regression analysis. Variables with p-values <0.1 were considered for multivariate analysis.

(B) Multivariate Cox regression analysis.

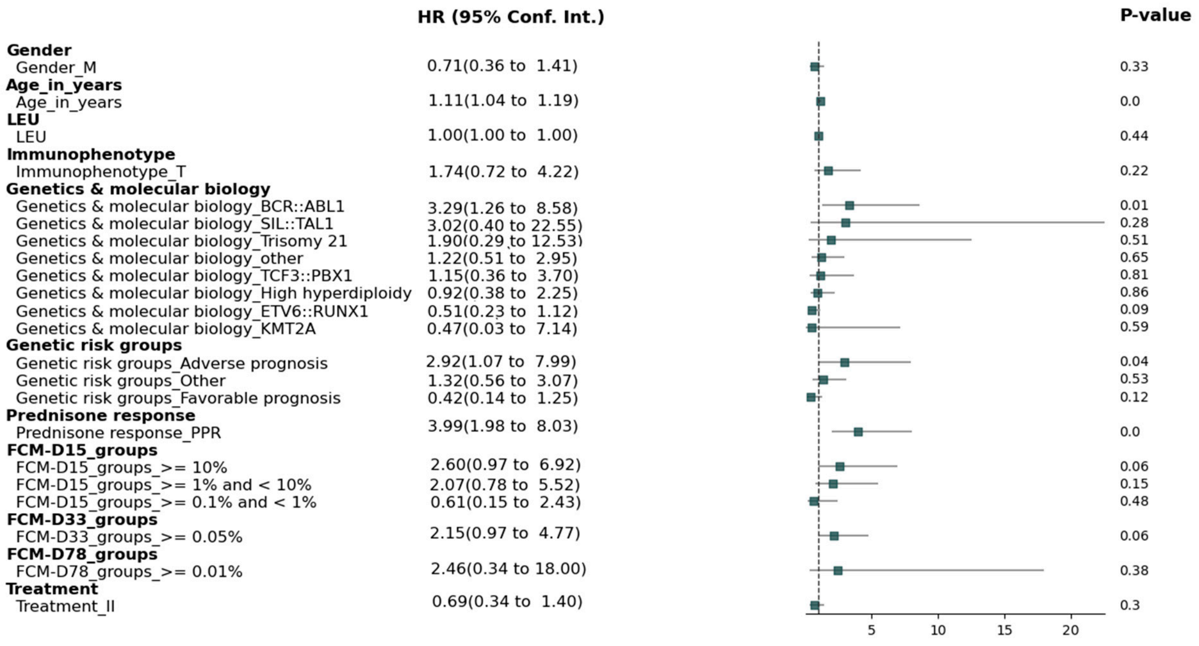

(A)

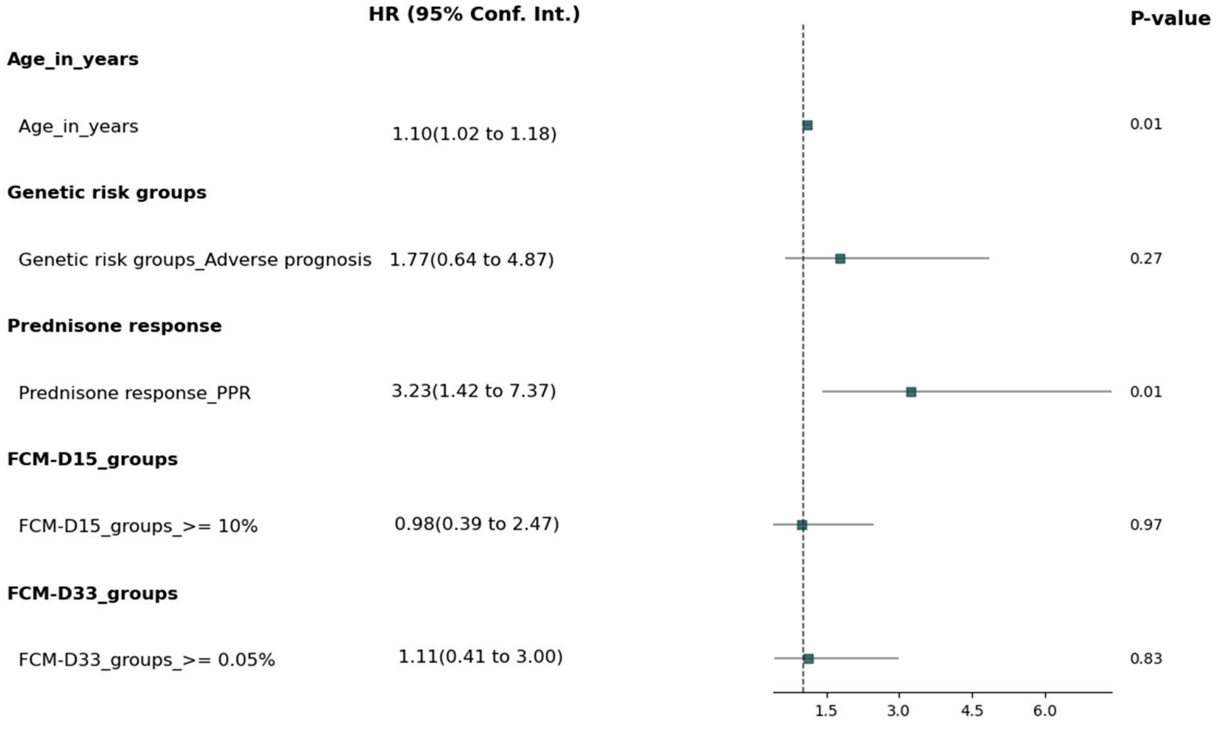

(B)

Figure S6

Figure S7. Forest plot illustrating Cox regression analysis on EFS.

(A) Univariate Cox regression analysis. Variables with p-values <0.1 were considered for multivariate analysis.

(B) Multivariate Cox regression analysis.

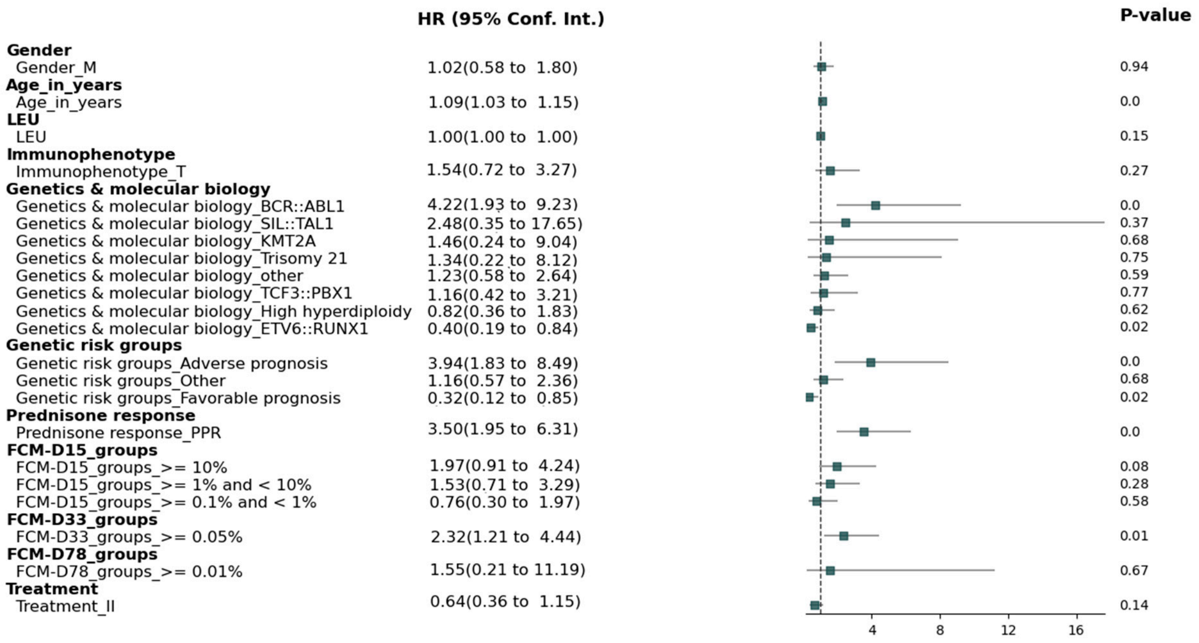

(A)

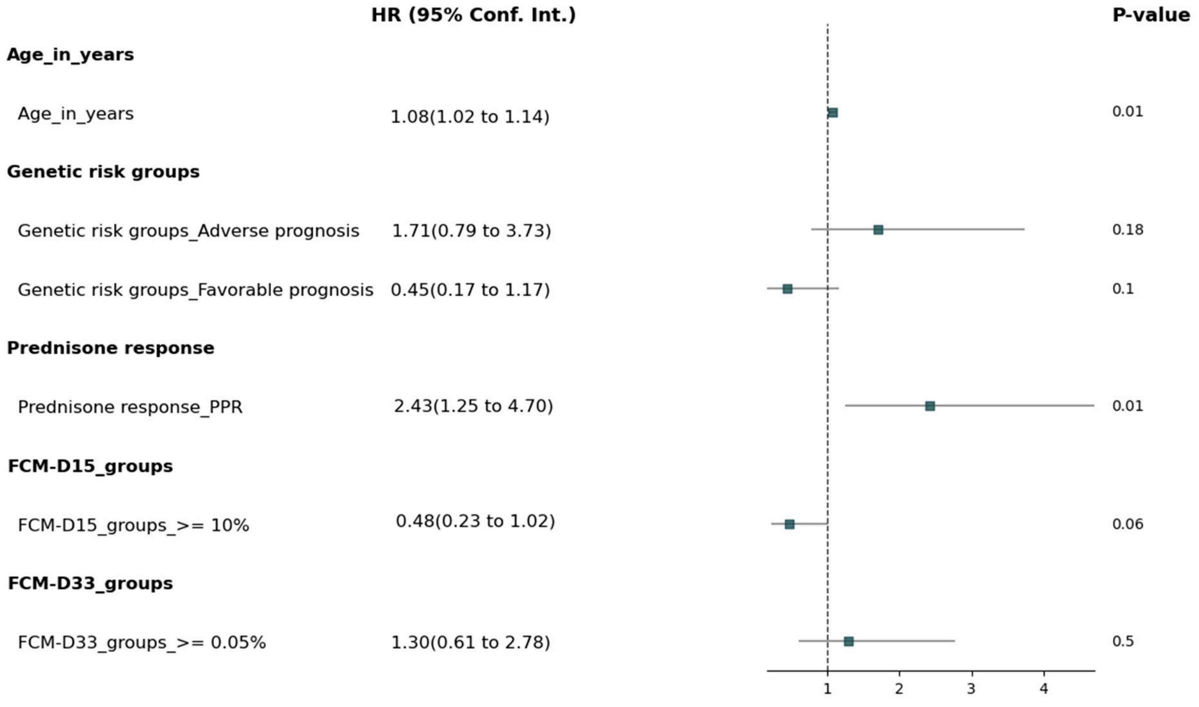

(B)

Figure S7
